# UBE2J2 promotes hepatocellular carcinoma cell epithelial-mesenchymal transition and invasion *in vitro*

**DOI:** 10.18632/oncotarget.17601

**Published:** 2017-05-03

**Authors:** Shaopeng Chen, Ying Tan, Haihua Deng, Zhifa Shen, Yanhong Liu, Pan Wu, Chunyan Tan, Yuyang Jiang

**Affiliations:** ^1^ Graduate School at Shenzhen, Tsinghua University, Shenzhen 518055, China; ^2^ School of Pharmaceutical Sciences, Tsinghua University, Beijing, 100084, P. R. China; ^3^ Key Laboratory of Laboratory Medicine, Ministry of Education of China, Zhejiang Provincial Key Laboratory of Medical Genetics, School of Laboratory Medicine and Life Sciences, Wenzhou Medical University, Wenzhou 325035, China; ^4^ Fuyong Hospital, Shenzhen, 518055, China

**Keywords:** hepatocellular carcinoma, UBE2J2, epithelial-mesenchymal transition, EGFR, invasion

## Abstract

Ubiquitin-conjugating enzyme E2 J2 (UBE2J2) is an ubiquitin proteasome component that responds to proteotoxic stress. We found that UBE2J2 was highly expressed in cellular protrusions of HCCLM3 metastatic hepatocellular carcinoma (HC) cells. Immunohistochemical analyses showed that UBE2J2 was expressed at higher levels in HC patient tissues than in corresponding non-tumor tissues. Because cellular protrusions are important for cell invasion, we hypothesized that UBE2J2 promotes HC cell invasion. We used chip-based surface plasmon resonance (SPR) to assess possible mechanisms of UBE2J2-regulated HCCLM3 cell invasion. We found that p-EGFR interacted with UBE2J2, and this finding was confirmed by co-immunoprecipitation analysis. UBE2J2 overexpression activated endothelial-mesenchymal transition in the non-invasive SMMC7721 HC cell line, and promoted invasion. UBE2J2 silencing reduced HCCLM3 cell invasion and endocytosis, and downregulated p-EGFR expression. p-EGFR inhibition by lapatinib reduced UBE2J2-promoted cell invasion, suggesting p-EGFR is important for UBE2J2-mediated HCCLM3 cell invasion. These findings demonstrate that endocytosis by HC cells is closely related to invasion, and may provide new anti-HC therapeutic targets. UBE2J2 may also be a novel biomarker for clinical HC diagnosis.

## INTRODUCTION

Hepatocellular carcinoma (HC) is one of the most common cancers worldwide [1, 2]. Metastasis is the primary cause of cancer patient death [3], and HC diagnostic and therapeutic outcomes remain unsatisfactory [2]. Additional studies of the molecular mechanisms underlying HC cell metastasis, and new anti-HC therapeutic targets are urgently needed [2].

Cell invasion is closely related to tumor metastasis [4]. Before invasion, most malignant tumor cells undergo epithelial-mesenchymal transition (EMT) to detach from surrounding tissue [5, 6]. Biomarkers, including E-cadherin, snail, slug, vimentin, CLDN-1, and N-cadherin, can be used to detect EMT or MET (reverse process of EMT). Cellular protrusions can drive cancer cells to dissociate from their surroundings and penetrate into the vasculature [7]. Highly dynamic actins assembled by the Arp2/3 complex and formins, along with dynamin and cortactin, both membrane trafficking machinery components, reportedly accumulate in finger-like protrusions [8–10]. Direct RNA sequencing (DRS) analysis of hepatocellular carcinoma cell protrusions will enhance our understanding of liver tumor cell metastasis and invasion.

Most misfolded proteins and extracellular proteins internalized via endocytosis are degraded through the ubiquitin proteasome pathway [11]. Ubiquitin-conjugating enzyme E2 J2 (UBE2J2) is an ubiquitin proteasome component that responds to proteotoxic stress [12–13]. As an endoplasmic reticulum-localized ubiquitin-conjugating enzyme, UBE2J2 associates with ubiquitin ligases, including TEB4 [14], parkin [15], CHIP [16], and cIAP1 [17], to promote proteasomal degradation [18]. The UBE2J2 hydrophobic carboxyl terminus region reportedly stabilizes UBE2J2 and mediates insertion of proteins into the ER membrane [18]. UBE2J2 also interacts with c-IAP1 to support TNF-R2-mediated TRAF2 ubiquitination and degradation [17]. So far, little evidence has implicated UBE2J2 in cell invasion.

This study found that UBE2J2 was highly expressed in HCCLM3 cell protrusions. We investigated mechanisms of UBE2J2-regulated HCCLM3 cell invasion and the relationship between endocytosis and cell invasion, providing new insights into HC metastasis and invasion regulation.

## RESULTS

### UBE2J2 is highly expressed in HCCLM3 cell protrusions

**Figure 1:**
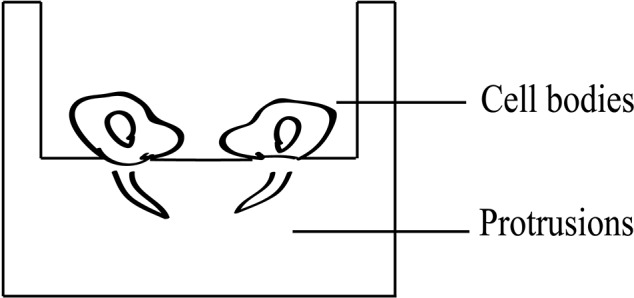
Cellular protrusion and cell body isolation HCCLM3 and SMMC7721 cells were seeded on invasion inserts with 1μm (diameter) pore membranes, allowing only protrusions to pass through. Twelve h after cells reached confluence, cellular protrusions and cell bodies were isolated using cell scrapers, and total RNA and proteins were collected.

We isolated cellular protrusions and cell bodies from HCCLM3 (a highly metastatic HC cell line) and SMMC7721 cells (a low metastatic HC cell line) via Boyden chamber isolation assay as described previously [19] (Figure 1). More than 7500 genes were sequenced using DRS and listed according their expression ratios in HPs (HCCLM3 protrusions) versus HBs (HCCLM3 cell bodies). Table 1 lists the 50 genes with the highest HP/HB expression ratios. PYCARD is related to inflammatory and apoptotic signaling pathways [20], and STXBP2, ATP6V0D1, and UBE2J2 are responsible for endocytosis [21–24]. Our DRS results suggest that endocytosis is related to HC cell metastasis and invasion.

**Table 1:** DRS of HCCLM3 and SMMC7721 cellular protrusion and cell bodies

Gene ID	Symbol	HP/HB	SP/SB	Reported on invasion
1382	CRABP2	2.51	/	√
10381	TUBB3	2.25	/	√
9022	CLIC3	2.24	/	√
7001	PRDX2	2.22	1.19	√
5499	PPP1CA	2.21	1.46	√
226	ALDOA	2.19	1.27	√
6237	RRAS	2.14	1.18	√
3217	HOXB7	2.11	1.06	√
6813	STXBP2	2.07	1.39	-
29108	PYCARD	2.07	/	-
9114	ATP6V0D1	2.07	1.36	-
71	ACTG1	2.01	/	√
256281	NUDT14	2.00	/	√
7086	TKT	1.96	1.43	√
3880	KRT19	1.91	/	√
5730	PTGDS	1.90	/	√
1397	CRIP2	1.90	/	√
11040	PIM2	1.90	0.98	√
118424	UBE2J2	1.89	1.09	-
293	SLC25A6	1.89	1.29	-
2010	EMD	1.88	1.30	√
9168	TMSB10	1.88	1.42	√
10636	RGS14	1.88	/	√
991	CDC20	1.87	1.23	√
26099	SZRD1	1.85	1.29	-
4282	MIF	1.85	1.30	√
51282	SCAND1	1.84	0.98	-
113452	TMEM54	1.84	/	-
7284	TUFM	1.83	1.37	-
79629	OCEL1	1.82	1.27	-
7283	TUBG1	1.82	1.13	√
3611	ILK	1.82	1.31	√
5003	SLC22A18AS	1.82	/	-
1465	CSRP1	1.81	1.33	√
10749	KIF1C	1.81	1.32	√
5696	PSMB8	1.81	1.45	-
55365	TMEM176A	1.81	/	-
6187	RPS2	1.80	/	-
217	ALDH2	1.80	1.25	-
10238	DCAF7	1.79	1.28	-
84885	ZDHHC12	1.79	1.33	-
7280	TUBB2A	1.79	/	√
27338	UBE2S	1.78	1.22	√
9235	IL32	1.78	1.01	√
3669	ISG20	1.77	/	-
1984	EIF5A	1.77	1.21	√
1616	DAXX	1.76	1.02	√
6920	TCEA3	1.76	1.26	-
6015	RING1	1.75	0.97	√
92170	MTG1	1.75	/	-

Abbreviations: HP: HCCLM3 protrusions; HB: HCCLM3 cell bodies; SP: SMMC7721 protrusions; SB: SMMC7721 cell bodies; “/”: gene could not be detected; “-”: little report of the given gene with respect to cell invasion; “√”: reports of the given gene with respect to cell invasion.

**Figure 2:**
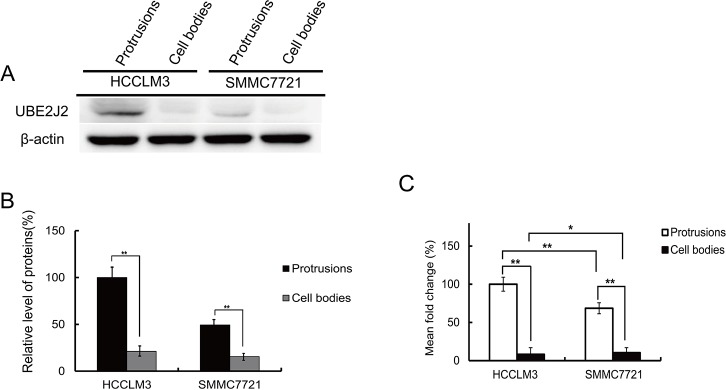
UBE2J2 expression in HCCLM3 and SMMC7721 cell protrusions and cell bodies **(A)** Western blot analysis of UBE2J2 in HCCLM3 and SMMC7721 cell protrusions and cell bodies. β-actin was used as the loading control. **(B)** Densitometric analysis. Results are shown with respect to control. **(C)**Cellular protrusion and cell body mRNA was quantified by RT-qPCR. *GAPDH* was used as an internal control. *P<0.05, **P<0.01 vs. control.

Protein lysates were analyzed using western blotting (Figure 2A–2B). UBE2J2 was more highly expressed in HCCLM3 cell protrusions than in SMMC7721 cells and cell bodies. RT-qPCR results revealed that *UBE2J2* transcription rates were greater in HCCLM3 protrusions than in cell bodies and SMMC7721 cells (Figure 2C).

### UBE2J2 is enriched in tumor tissues

**Figure 3:**
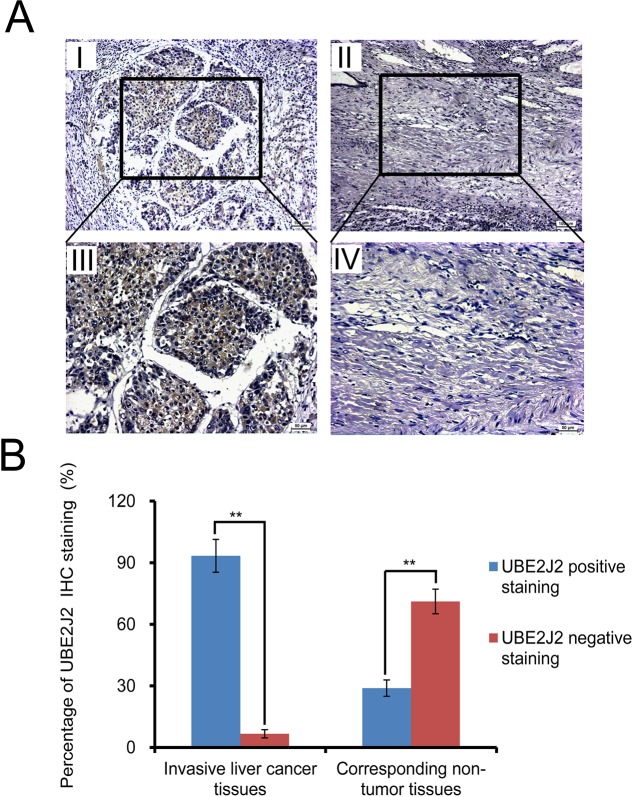
IHC analysis of UBE2J2 in invasive HC and corresponding non-tumor tissues **(A)** Paraffin-embedded metastasized HC tissue sections were stained with DAB. HC tissues (I. & III.) mostly stained positive for UBE2J2 (score >2). Positive staining was infrequent in corresponding non-tumor tissues (II. & IV.). Scale bar = 100 μm for (I, II), 50μm for (III, IV). **(B)** Statistical analysis of UBE2J2 staining in HC patient tissues. **P<0.01.

Forty-five invasive HC and corresponding non-tumor tissue samples were analyzed immunohistochemically (Figure 3). Staining results were scored and classified using the five-point scoring system as described [25]. Positive UBE2J2 staining (score >2) was detected in 42/45 (93.3%) metastatic HC samples, and 13/45 (28.9%) corresponding non-tumor tissues, (*P*<0.01, Figure 3). These results show that UBE2J2 is highly expressed in metastatic HC tissues.

### UBE2J2 knockdown decreases HCCLM3 cell invasion

**Figure 4:**
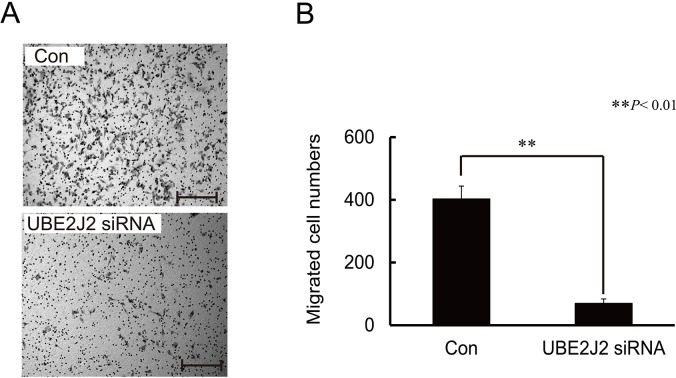
Effects of UBE2J2 knockdown on HCCLM3 cell invasion HCCLM3 cells were transfected with UBE2J2 or scramble siRNA. Cells were seeded on Matrigel-coated chambers 24 h post-transfection. **(A)** Invaded cells were stained with 0.08% trypan blue. **(B)** Cells were counted in five random fields. Scale bar = 200 μm. **P<0.01.

UBE2J2 was silenced using specific siRNAs in HCCLM3 cells, and cell invasion was measured using the Boyden chamber assay. The number of migrated cells was lower in UBE2J2-silenced cells than in controls (Figure 4), indicating that UBE2J2 regulates HCCLM3 cell invasion.

### UBE2J2 knockdown induces MET in HCCLM3 cells

**Figure 5:**
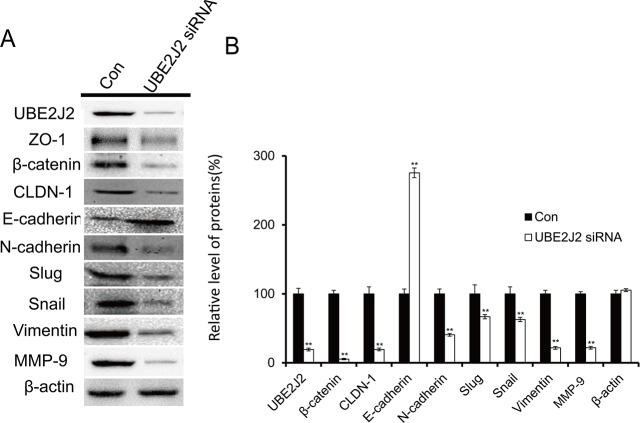
Effects of UBE2J2 knockdown on HCCLM3 cell MET **(A)** Western blot analysis of UBE2J2, ZO-1, β-catenin, CLDN-1, E-cadherin, N-cadherin, slug, snail, vimentin, and MMP-9 levels. β-actin was used as loading control. **(B)** Densitometric analysis. Results are shown with respect to control. **P<0.01.

EMT/MET biomarkers were used to detect the effects of UBE2J2 on the EMT/MET processes via western blotting. UBE2J2 knockdown downregulated ZO-1, β-catenin, CLDN-1, N-cadherin, slug, snail, MMP-9, and vimentin by 80.7%, 94.6%, 80.7%, 59.4%, 33.1%, 37.1%, 78.1%, and 78.3%, respectively (Figure 5). E-cadherin expression was upregulated by 175.4% (Figure 5). These results suggest that UBE2J2 knockdown induces MET in HCCLM3 cells.

### UBE2J2 overexpression activates SMMC7721 cell EMT and promotes invasion

**Figure 6:**
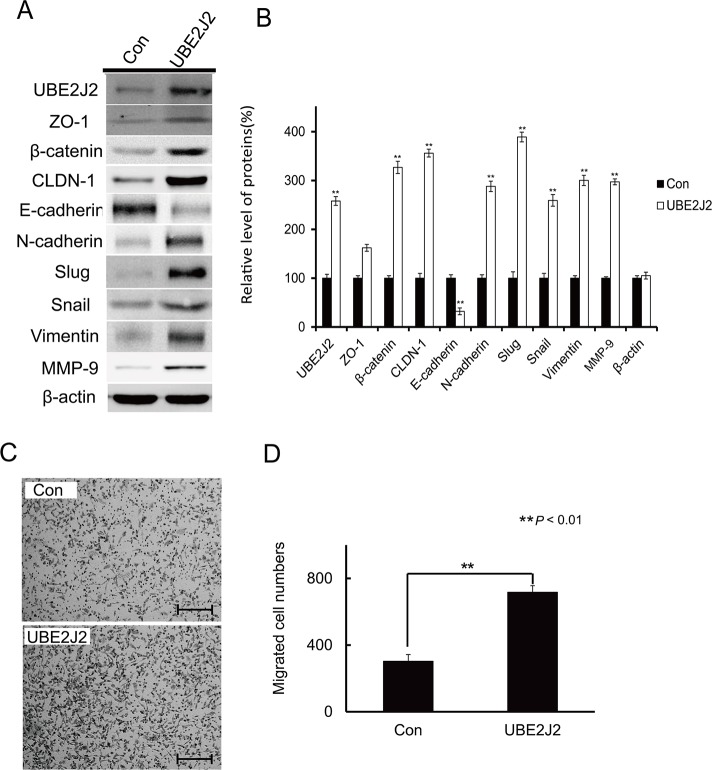
Effects of UBE2J2 overexpression on SMMC7721 cell EMT and invasion **(A)** Western blot analysis of EMT biomarker levels. β-actin was used as loading control. **(B)** Densitometric analysis. Results are shown with respect to control. Cells that invaded through the membrane were stained with 0.08% trypan blue **(C)** and counted **(D)**. Scale bar represents 200 μm. **P<0.01.

An UBE2J2 expression plasmid was transfected into SMMC7721 cells. After 24 h, cell lysates were collected and analyzed via western blotting using EMT biomarker antibodies to examine the effects of UBE2J2 on EMT (Figure 6A). UBE2J2 overexpression (by 158%), β-catenin, CLDN-1, N-cadherin, slug, snail, vimentin, MMP-9, and ZO-1 levels increased by 226.89%, 256.15%, 188.23%, 289.25%, 159.34%, 200.29%, 197.3%, and 62.07%, respectively (Figure 6B). E-cadherin expression was reduced to 32.16% (Figure 6B). These results suggest that UBE2J2 overexpression induced EMT in SMMC7721 cells. UBE2J2 overexpression also resulted in higher numbers of migrated cells (735±32) than controls (343±28) (Figure 6C).

### UBE2J2 binds p-EGFR

**Figure 7:**
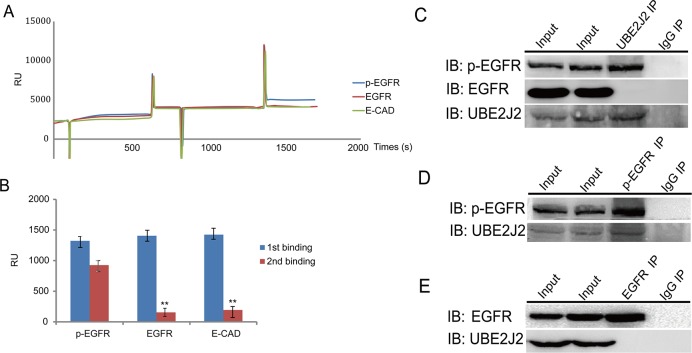
UBE2J2 and p-EGFR SPR and Co-IP analyses **(A)** SPR analysis by Biacore X100.Anti-UBE2J2 antibody was immobilized onto a CM5 chip, followed by cell lysate protein binding and various antibodies. **(B)** SPR binding quantification. **(C)** The p-EGFR-UBE2J2 complex was pulled down by anti-UBE2J2, anti-p-EGFR, and anti-EGFR antibodies, and examined via immunoblotting. IgG was used as negative control.

Chip-based surface plasmon resonance (SPR) was used to screen for potential UBE2J2-associating proteins. SPR biosensors monitor refractive index (measured in resonance units, RU) alterations caused by binding of analytes to ligands immobilized on sensor chips [26]. Anti-UBE2J2 antibody was immobilized on CM5 sensor chips. RU values increased from 2300 to 3800 when cell-extracted proteins bound the immobilized antibody (Figure 7A). An RU increase from 3800 to 5100 indicated that p-EGFR bound UBE2J2 (Figure 7A–7B). Co-IP analysis results further confirmed that p-EGFR binds UBE2J2 (Figure 7C–7E).

### UBE2J2 and p-EGFR were enriched in HC tissues

**Figure 8:**
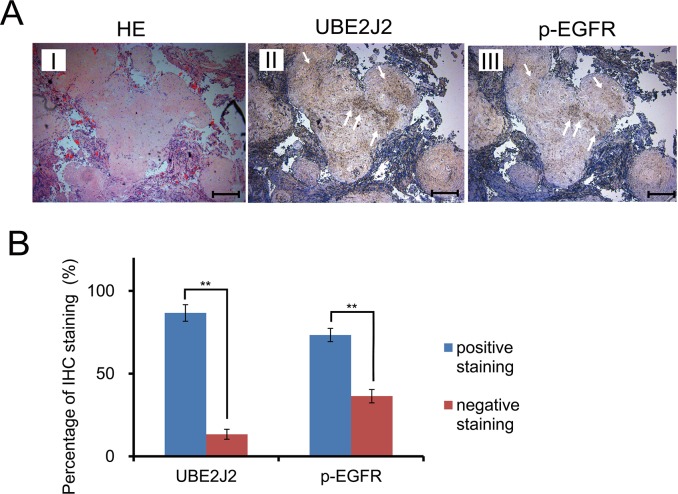
UBE2J2 and p-EGFR expression in invasive HC serial sections **(A)** Paraffin-embedded metastasized HC tissue serial sections were stained with H&E and DAB. H&E staining cells (I). UBE2J2 staining cells (II). p-EGFR staining cells (III). Scale bar = 250 μm. **(B)** Statistical analysis of UBE2J2 and p-EGFR positive staining. **P<0.01.

Serial sections of metastasized HC tissues were stained using anti-UBE2J2 and anti-p-EGFR antibodies (Figure 8A). UBE2J2-positive and p-EGFR-positive staining was observed in 86.7% (13/15) and 73.3% (11/15) of samples, respectively (Figure 8B, *P*<0.01), indicating that p-EGFR and UBE2J2 were highly expressed in HC tissues. The highest-scoring UBE2J2 and p-EGFR positive staining locations are indicated by white arrows (Figure 8A).

### UBE2J2 knockdown downregulates p-EGFR

**Figure 9:**
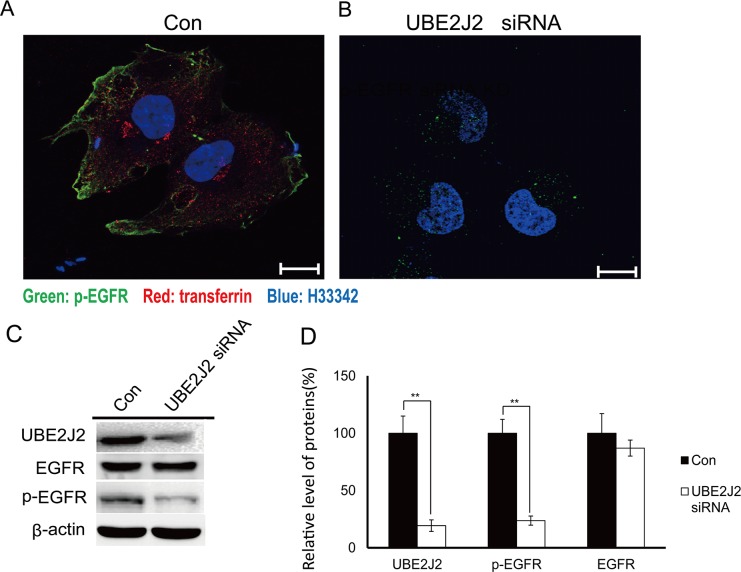
p-EGFR expression in UBE2J2-silenced HCCLM3 cells HCCLM3 cells were transfected with scramble **(A)** or UBE2J2 siRNA **(B)** for 24 h. Cells were starved for 3 h, treated with transferrin (5mg/ml, red) for 30 min, and then stained with anti-p-EGFR antibody (green) and H33342 (blue). Scale bars = 5 μm.**(C)** UBE2J2 and p-EGFR western blot analysis. **(D)** Densitometric analysis. Results are shown with respect to control. **P<0.01.

UBE2J2-knockdown HCCLM3 cells were immuno-fluorescently stained 24 h post-transfection. p-EGFR was downregulated in treated cells compared with controls (Figure 9A). Transferrin, an endocytosis indicator, was also downregulated in UBE2J2-silenced cells (Figure 9B). Western blotting results showed that UBE2J2 knockdown reduced p-EGFR expression (Figure 9C) to 24% of control levels (Figure 9D). These results together indicate that UBE2J2 promotes p-EGFR expression and endocytosis.

### Lapatinib attenuates UBE2J2-mediated cell invasion

**Figure 10:**
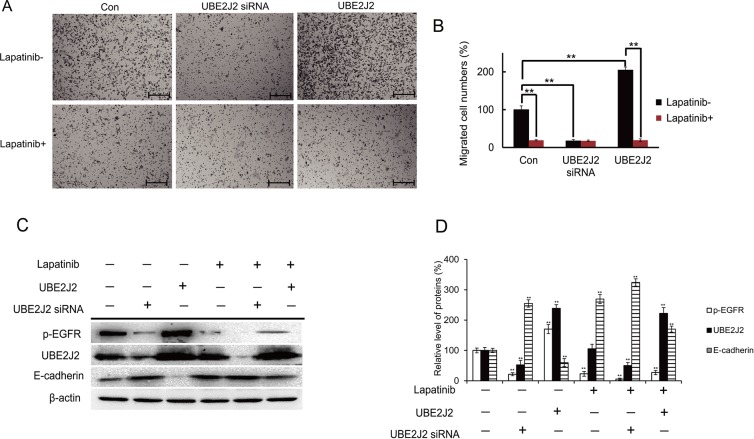
Effects of p-EGFR on UBE2J2-mediated HCCLM3 cell invasion HCCLM3 cells were treated with vehicle or Lapatinib (10 nM) for 24 h, followed by UBE2J2 knockdown or overexpression. **(A)** Cell invasion as assessed via Boyden chamber assay. **(B)** Migrated cells were counted. Scale bar = 200 μm. **(C)** Western blot analysis of the indicated proteins. **(D)** Densitometric analysis. Results are shown with respect to control. **P<0.01.

Lapatinib (10 nM), a p-EGFR inhibitor [27], was used to explore the role of p-EGFR in UBE2J2-regulated cell invasion. Lapatinib-mediated p-EGFR suppression attenuated the effects of UBE2J2 on cell invasion (Figure 10A–10B). Western blotting confirmed that Lapatinib suppressed p-EGFR, resulting in E-cadherin downregulation (Figure 9C). p-EGFR inhibition also reduced the suppression on E-cadherin by UBE2J2 (Figure 10C). These results together suggest that UBE2J2-regulated HCCLM3 cell invasion depends on p-EGFR.

## DISCUSSION

Cellular protrusions are highly dynamic structures involved in cell invasion [9]. To investigate how cellular protrusions promote invasion, mRNA from HCCLM3 cell protrusions and cell bodies was analyzed by direct RNA sequencing (DRS). Of the first four genes in these results not yet associated with cell invasion, three (ATP6VOD1, STXBP2, and UBE2J2) are important for endocytosis [21–24].

Endocytosis negatively regulates cell–extracellular environment interactions and controls cell signaling, for example through EGFR degradation by mono-ubiquitination of receptor tyrosine kinases (RTKs) [28, 29], Ras activation [30, 31], caveolae-mediated TGF-β receptor attenuation [32], and clathrin-dependent internalization [33]. Recent work has related endocytosis with cell invasion and motility. WNT5A promoted cancer cell invasion in a receptor-mediated endocytosis-dependent manner [34]. Clathrin-mediated endocytosis is required for EGF-directed chemotactic invasion by MDA-MB-231 cells [34] and *Tannerella forsythia* invasion in oral epithelial cells [35]. Integrin endocytosis is required for αvβ6-mediated carcinoma cell migration and invasion [36]. Based on these findings and our DRS results, we hypothesized that ATP6VOD1, STXBP2, and UBE2J2 might regulate HC cell invasion and metastasis.

Western blotting and RT-qPCR analyses showed that UBE2J2 was highly expressed in HCCLM3 cell protrusions. STXBP2 and ATP6V0D1 were also highly expressed (data not shown here). IHC analyses showed UBE2J2-positive staining in most HC tissues compared with corresponding non-tumor tissues (Figure 3), indicating that UBE2J2 might be a useful biomarker for HC diagnosis. UBE2J2 was silenced in HCCLM3 cells using specific siRNA, and cell invasion was measured via Boyden chamber assay. Invasion was decreased in silenced cells, indicating that UBE2J2 regulates HCCLM3 cell invasion.

The EMT-MET switch is fundamental to tumor metastasis [37]. EMT allows cancer cells in the primary tumor site to break through the basement membrane and enter the bloodstream through intravasation [38]. Invasive tumor cells that survive this process usually undergo MET within the new environment. Because UBE2J2 promotes HCCLM3 cell invasion, we assessed whether or not UBE2J2 knockdown might induce MET in these cells. We found that numerous MET biomarkers, including β-catenin, CLDN-1, N-cadherin, slug, snail, vimentin, ZO-1, MMP-9, were downregulated following UBE2J2 knockdown, and E-cadherin was upregulated. UBE2J2 overexpression in non-invasive SMMC7721 cells appeared to induce EMT and cell invasion, indicating that UBE2J2 regulates the EMT-MET switch.

To explore potential mechanisms of UBE2J2-regulated HCCLM3 cell invasion, we screened for UBE2J2-interacting proteins using chip-based SPR. Numerous cell invasion-related protein antibodies were tested, but only p-EGFR bound UBE2J2. p-EGFR controls cell invasion via AKT and MMPs [39, 40]. We assessed HC cell invasion and protein levels following UBE2J2 silencing or p-EGFR inhibition. While p-EGFR levels appeared dependent on UBE2J2 expression, p-EGFR inhibition reduced UBE2J2-promoted HCCLM3 cell invasion. Our findings indicate that UBE2J2 binds p-EGFR to promote HCCLM3 cell invasion.

**Figure 11:**
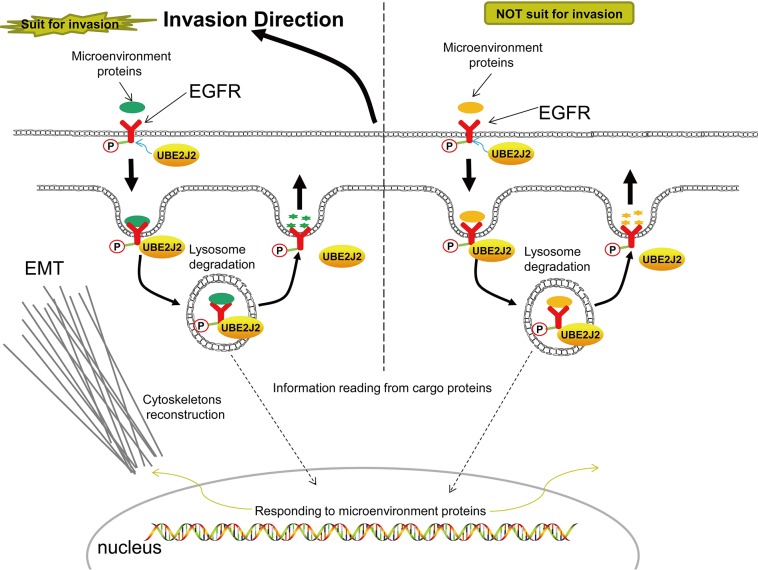
Proposed mechanism of UBE2J2-mediated HCCLM3 cell invasion and endocytosis

Transferrin is an endocytosis indicator [41]. We found that UBE2J2 knockdown suppressed transferrin endocytosis. Because UBE2J2 also promotes HC cell invasion, we hypothesize that endocytosis is closely related to invasion. Protein ubiquitin (UB) modification is an important aspect of endocytosis [42]. Proteins secreted by other cells are captured by cell membrane receptors, internalized via endocytosis, sorted, and degraded by proteases in the lysosome [12, 42]. Our study confirmed that UBE2J2 positively regulates HC cell endocytosis. p-EGFR reportedly stabilizes snail and slug to trigger EMT and tumor metastasis [43]. We showed that UBE2J2 binds p-EGFR to promote HC cell invasion and EMT. The UBE2J2 hydrophobic carboxyl terminus anchors to the ER membrane and associates with ubiquitin ligases to degrade cargo proteins in the lysosome [44]. Based on information from the microenvironment, cells make adjustments, such as triggering EMT or MET, reorganizing actin and tubulin, reconstructing cytoskeletons, and migrating (Figure 11).

In conclusion, the p-EGFR-UBE2J2 complex appears to promote HCCLM3 cell invasion and endocytosis. Our findings demonstrate that endocytosis in HC cells is closely related to invasion, and may provide new anti-HC therapeutic targets. UBE2J2 may also be a novel biomarker for clinical HC diagnosis.

## MATERIALS AND METHODS

### Cell culture

Cell culture supplies were purchase from Life Technologies (Carlsbad, USA) and Corning (New York, USA). Human liver cancer cell lines, HCCLM3 and SMMC7721, were bought from the cell bank at the Chinese Academy of Sciences. Cells were grown in DMEM supplemented with 10% FBS, in an incubator with 5% CO_2_ at 37°C.

### Protrusion isolation and direct RNA sequencing (DRS)

Cellular protrusion and cell body mRNA was extracted as described [19]. Cells were seeded on invasion inserts with 1μm pore membranes (from BD Company). Twelve h after cells reached confluence, cellular protrusions were cut using a cell scraper, and mRNA was extracted using TRIzol. DRS was conducted via BGI RNA-Seq (Quantification) Analysis (BGI Tech, Shenzhen, China). The experiment was repeated three times and genes not detected at any time were excluded. More than 7,500 genes were analyzed and listed according to the ratio of expression in protrusions (HP) versus cell bodies (HB).

### Cell invasion assay

Cell invasion assays were performed as described [45, 46]. Invasion inserts with 8μm pore membranes (Corning, New York, USA) were coated with fibronectin (Sigma-Aldrich, Missouri, USA) as described [47]. Cells were pretreated with a specific or control siRNA for 6 h. Pretreated cells were seeded onto inserts to reach confluence in 12 h, and then cultured for another 24 h. After fixation with 4% formaldehyde, non-invading cells on the upper sides of membranes were removed by cotton swab. Invading cells were stained with 0.08% trypan blue for 15 min as described [46] and photographed using a bright-field light microscope. Cells were counted in five random fields using Image-Pro Plus 6.0 from Media Cybernetics (MD, USA).

### RT-qPCR

Cells were treated with specific or control siRNA for 24 h and total RNA was extracted using RNAzol as described [19]. cDNA was obtained using the PrimeScriptTM RT Reagent Kit (Takara, Dalian, China). *UBE2J2* was amplified by RT-PCR using the KAPA SYBR Fast qPCR Kit (MA, USA) and measured using the 7500 Fast Real-Time PCR System (ABI, Massachusetts, USA). The following primers were used: *UBE2J2* forward: 5′-TCTGTCTCCACCATCCTGACTG-3′, reverse: 5′-GCTAAACTCTGCACTGCCAGTTG-3′; *GAPDH* forward: 5′-GTCTCCTCTGACTTCAACAGCG-3′, reverse: 5′-ACCACCCTGTTGCTGTAGCCAA-3′. Primers were synthesized by Life Technologies (Massachusetts, USA). Gene expression was analyzed using the 2^−ΔΔCt^ method as described [48].

### Western blotting

Cells were lysed in RIPA buffer to extract whole-cell proteins [49]. Protein concentrations were measured using the DC Protein Assay Kit I (Bio-Rad, California, USA). Equal amounts of protein were subjected to 10% SDS polyacrylamide gel electrophoresis, and then transferred to PVDF membranes. The following primary antibodies were used: UBE2J2 (Millipore, Massachusetts, USA), EMT antibody kit, EGFR, and p-EGFR (Tyr1068) (Cell Signaling, Massachusetts, USA). Horseradish peroxidase-conjugated secondary antibody signals were detected and measured using the Luminescent Image Analyzer Tanon 5200 (Shanghai, China). Band densities were measured via ImageQuant software (Molecular Dynamics, Sunnyvale, CA, USA), and expressed as percentage of the β-actin band density.

### siRNA-mediated gene silencing

siRNA oligos were designed and synthesized by GenePharma Company. Specific siRNAs included: UBE2J2: 5′-CCAGAGAAUUUCCUUUCAATT-3′ and 5′-UUGAAAGGAAAUUCUCUGGTT-3′; negative control: 5′-UUCUCCGAACGUGUCACGUTT-3′ and 5′-ACGUGACACGUUCGGAGAATT-3′; GAPDH positive control: 5′-UGACCUCAACUACAUGGUUTT-3′ and 5′-AACCAUGUAGUUGAGGUCATT3′. siRNA oligos were co-transfected into cells with Lipofectamine 2000 as described [19]. Cell lysates were analyzed using western blotting and RT-qPCR. Invasion rates of cells treated with specific or control siRNAs were measured via Boyden chamber assay. ANOVA was performed to evaluate differences between controls and siRNA knockdown groups, followed by Tukey post hoc analysis using Prism (GraphPad).

### *UBE2J2* cloning and overexpression

*UBE2J2* plasmid was constructed by cloning the *UBE2J2* PCR amplicon with specific primers into the XbaI site in the pEGFP-N3 plasmid (Clontech). *UBE2J2* forward cloning primer: 5′-GCTCTAGAGCATGACCCCTTATGAAGGTGG-3′, and reverse: 5′-GCTCTAGAGCTCACTCCTGCGCGATGCT-3′. After ligation, the amplicon was transfected into DH5α competent cells followed by plasmid extraction and transient transfection of *UBE2J2* into SMMC7721 cells using Lipofectamine 2000 as described [20]. After 24 h transfection, cells were subjected to Boyden chamber assay to assess cell invasion. Cell lysates were analyzed via western blotting with EMT biomarker antibodies to examine the effects of UBE2J2 on EMT.

### Immunohistochemistry (IHC)

Forty-five paraffin-embedded hepatoma and corresponding non-tumor tissue sections were obtained from Shanghai Outdo Biotech Company (Shanghai, China) and Fuyong hospital (Shenzhen, China). Sections were deparaffinized using dimethylbenzene, 100% ethanol, 90% ethanol, 80% ethanol, and 70% ethanol, followed by EDTA antigen retrieval and blocking. EDTA antigen retrieval solution was purchased from Beyotime (Suzhou, China) and the double standard blockers were from JianCheng Bioengineering Institute (Nanjing, China). Tissue sections were incubated with primary antibodies (1:200), washed, incubated with secondary antibodies (1:200), and then stained using 3,3′-Diaminobenzidine (DAB; JianCheng Bioengineering Institute, Nanjing, China) and hematoxylin (BaSo Company, Taiwan). Stained sections were analyzed and classified using a 5-point scoring system as described [25]. A score >2 points was considered positive staining. For serial section IHC staining, serial sections from 15 invasive HCs (provided by Fuyong Hospital, Shenzhen, China) were stained with hematoxylin and eosin (H&E), and anti-UBE2J2, and anti-p-EGFR antibodies.

### Chip-based surface plasmon resonance (SPR)

UBE2J2-associated proteins were screened using a Biacore X100 SPR sensor (Biacore, Uppsala, Sweden) as described [50]. Anti-UBE2J2 antibody and normal IgG were immobilized to a CM5 research-grade sensor chip (carboxymethyldextran-derivatized surface) using the standard EDC/NHS [N-ethyl-N”-(dimethylaminopropyl) carbodiimide/N-hydroxysuccinimide] coupling method [51]. The CM5 Chip was blocked with 3% BSA to prevent nonspecific binding. Cells were lysed using RIPA buffer. Proteins were pre-concentrated using an ultrafiltration tube (Milipore), and then injected into the microfluidic system. After washing with HEPES buffer (50 mM HEPES, pH 7.4, 150 mM NaCl), UBE2J2 and any associated proteins remained on the CM5 Chip surface. β-catenin, CLDN-1, N-cadherin, E-Cadherin, slug, snail, vimentin, EGFR, p-EGFR, MMP-9, and MMP-2 antibodies were used to detect these cell invasion-related proteins. The process was automated using control software for the Biacore X100.

### Immunofluorescence staining

HCCLM3 cells were cultured in glass bottom dishes and transfected with UBE2J2 siRNA for 24 h using Lipofectamine 2000 as described [19]. After 3 h of serum-free starvation, cells were treated with transferrin (20ug/ml) for 30 min. Cells were fixed with 4% formaldehyde for 15 min and permeabilized with 0.25% Triton X-100 [52]. Cells were incubated with p-EGFR primary and Dylight 488 Donkey Anti-Rabbit secondary antibodies as described [53, 54]. Cells were examined using an Olympus confocal laser-scanning microscope and images were analyzed using IPP6.0 (Image-Pro Plus 6.0).

### Statistical analysis

Data are expressed as means ± standard deviation (SD) of three independent experiments, and were analyzed with SPSS software using Student's t test. *P*<0.05 was considered statistically significant.
